# Foot and Ankle Care by Podiatrists and Amputations in Patients With Diabetes and Kidney Failure

**DOI:** 10.1001/jamanetworkopen.2024.0801

**Published:** 2024-03-01

**Authors:** Tze-Woei Tan, Bryan Caldwell, Yi Zhang, Onkar Kshirsagar, Dennis J. Cotter, Thomas W. Brewer

**Affiliations:** 1 Keck School of Medicine of University of Southern California, Los Angeles; 2 Kent State University College of Podiatric Medicine, Independence, Ohio; 3 Medical Technology and Practice Patterns Institute, Bethesda, Maryland; 4 Kent State University College of Public Health, Kent, Ohio

## Abstract

**Importance:**

Patients with kidney failure have an increased risk of diabetes-related foot complications. The benefit of regular foot and ankle care in this at-risk population is unknown.

**Objective:**

To investigate foot and ankle care by podiatrists and the outcomes of diabetic foot ulcers (DFUs) in patients with kidney failure.

**Design, Setting, and Participants:**

This retrospective cohort study included Medicare beneficiaries with type 2 diabetes receiving dialysis who had a new DFU diagnosis. The analysis of the calendar year 2016 to 2019 data from the United States Renal Data System was performed on June 15, 2023, with subsequent updates on December 11, 2023.

**Exposures:**

Foot and ankle care by podiatrists during 3 months prior to DFU diagnosis.

**Main Outcomes and Measures:**

The outcomes were a composite of death and/or major amputation, as well as major amputation alone. Kaplan-Meier analysis was used to estimate 2 to 3 years of amputation-free survival. Foot and ankle care by podiatrists and the composite outcome was examined using inverse probability-weighted Cox regression, while competing risk regression models were used for the analysis of amputation alone.

**Results:**

Among the 14 935 adult patients with kidney failure and a new DFU (mean [SD] age, 59.3 [12.7] years; 35.4% aged ≥65 years; 8284 men [55.4%]; Asian, 2.7%; Black/African American, 35.0%; Hispanic, 17.7%; White, 58.5%), 18.4% (n = 2736) received care by podiatrists in the 3 months before index DFU diagnosis. These patients were older, more likely to be male, and have more comorbidities than those without prior podiatrist visits. Over a mean (SD) 13.5 (12.0)-month follow-up, 70% of those with podiatric care experienced death and/or major amputation, compared with 74% in the nonpodiatric group. Survival probabilities at 36 months were 26.3% vs 22.8% (*P* < .001, unadjusted Kaplan-Meier survival analysis). In multivariate regression analysis, foot and ankle care was associated with an 11% lower likelihood of death and/or amputation (hazard ratio [HR], 0.89 95% CI, 0.84-0.93) and a 9% lower likelihood of major amputation (above or below knee) (HR, 0.91; 95% CI, 0.84-0.99) than those who did not.

**Conclusions and Relevance:**

The findings of this study suggest that patients with kidney failure at risk for DFUs who receive foot and ankle care from podiatrists may be associated with a reduced likelihood of diabetes-related amputations.

## Key Points

**Question:**

Is preemptive foot and ankle care provided by podiatrists associated with a reduced risk of limb loss and/or mortality in patients with kidney failure receiving dialysis who are at risk of developing diabetic foot ulcers?

**Findings:**

In this cohort study including 14 935 patients aged 40 years or older with kidney failure, foot and ankle care by podiatrists prior to the occurrence of a diabetic foot ulcer was associated with lower rates of amputation and/or death. The rate of major amputation (above-knee, below-knee) was also decreased.

**Meaning:**

The findings of this study suggest that, for individuals with kidney failure receiving dialysis and susceptible to diabetes-related limb loss, receiving foot and ankle care by podiatrists before the onset of diabetic foot ulcers may be associated with improved outcomes.

## Teaser

This cohort study examines the occurrence of limb loss and mortality in patients with diabetic foot ulcers who are undergoing dialysis who have vs have not received preventive care by podiatrists.

## Introduction

Foot ulceration is one of the most serious and costly complications of diabetes worldwide, including the US.^1^ Up to 34% of adults with diabetes develop diabetic foot ulcers (DFUs), and two-thirds of DFUs recur, even after healing.^1,2^ These foot ulcers precede 80% of lower extremity amputations and are associated with diminished physical function, reduced quality of life, and increased mortality risk.^3,4,5^ The co-occurrence of kidney insufficiency among individuals with DFUs increases the risk of peripheral artery disease (PAD), lower extremity amputation, and mortality.^6,7^ In one study, patients with kidney failure accounted for nearly one-third of amputations within the overall diabetes population.^8^

Among the population of patients with kidney failure, approximately 10% to 32% of those receiving hemodialysis have active DFUs, while 22% to 41% have had a history of such ulcers.^9,10^ The severity of kidney disease corresponds to DFU outcomes.^11,12^ Individuals undergoing kidney replacement therapy face a 5-fold increased risk of DFUs compared with those with chronic kidney disease (stage 4 or 5) not receiving dialysis.^13^ Moreover, a study by Game et al^14^ suggested the onset of dialysis therapy itself seems to amplify the risk of DFUs among individuals with diabetes. In addition, the impact of diabetic foot complications extends to health care use, with DFUs the leading cause of hospitalizations among patients with kidney failure.^8,11^ Collectively, the cost of annual cost of DFU care to Medicare is estimated to be $9 billion, and the cost to private insurers is $1.3 billion.^15^

Recognizing the heightened risk of DFUs and their adverse outcomes among patients with diabetes and kidney failure, the 2023 International Working Group on Diabetic Foot practice guidelines categorize these patients as high risk (category 3), regardless of a history of DFU or amputation.^16^ This classification underscores the necessity of regular foot care and screening. Podiatrists play an instrumental role in treating diabetic foot complications in the US.^17,18,19^ While previous research has reported the importance of foot and ankle care by podiatrists in preventing amputations and hospitalizations in those with DFUs, the specific impact of such preemptive care in patients with kidney failure and diabetes at high risk for DFUs remains unexplored.^17,20^

Using a comprehensive and nationally representative cohort of US patients undergoing dialysis, this study sought to elucidate whether the outcomes of DFUs differ between patients with kidney failure who had preemptive foot and ankle care by podiatrists within 3 months before the index DFU diagnosis and those who did not.

## Methods

### Data Sources

We queried the US Renal Data System claims data from 2016 to 2017 to identify Medicare beneficiaries with type 2 diabetes receiving dialysis with a new diagnosis of DFU in 2017.^21^ The US Renal Data System, a national repository of information on patients with kidney failure, was used for analysis (eMethods in Supplement 1).^22^ The University of Arizona Institutional Review Board exempted this study from review and the requirement for informed consent due to the use of deidentified data. We followed the Strengthening the Reporting of Observational Studies in Epidemiology (STROBE) reporting guideline.^23^

### Study Populations

**Figure 1.**
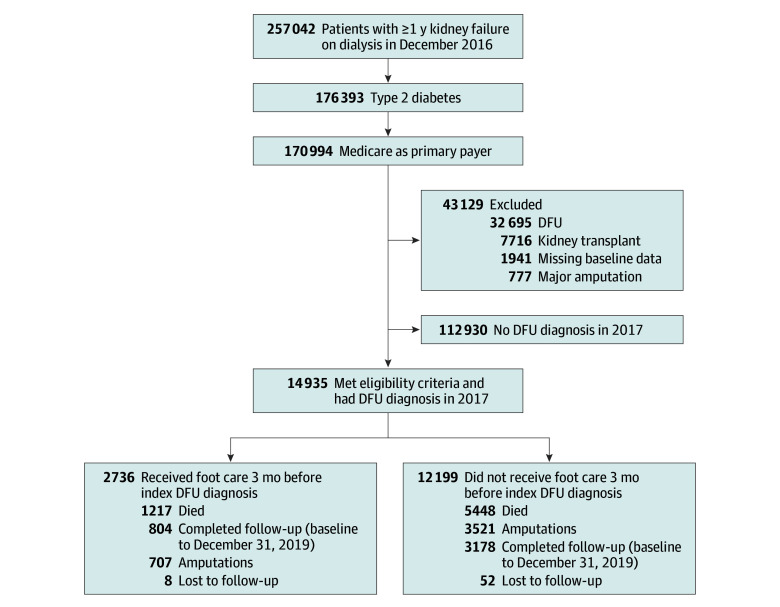
Flowchart of Study Cohort Selection DFU indicates diabetic foot ulcer.

Figure 1 illustrates the study cohort selection criteria. From an initial pool of 176 393 patients with type 2 diabetes and kidney failure undergoing dialysis in December 2016, we focused on Medicare beneficiaries to ensure sufficient claims history (n = 170 994). The index date was the first DFU diagnosis in 2017 (study baseline). We excluded patients with a prior diagnosis of DFU in 2016 (n = 32 695), history of major amputation (above or below the knee) (n = 777), kidney transplant (n = 7716), and incomplete baseline information (n = 1941). The final study cohort (n = 14 935) was followed up until December 31, 2019, for major amputation, death, or loss to follow-up (defined as missing Medicare claims in 60 consecutive days). Median follow-up time was 12 (IQR, 2-26) months, and mean (SD) follow-up time was 13.5 (12.0) months. A full list of related *International Statistical Classification of Diseases and Related Health Problems, 10th Revision, Clinical Modification* (*ICD-10-CM*) and Healthcare Common Procedure Coding System (HCPCS) procedures are provided in eTable 1 in Supplement 1.

### Outcomes

We assessed 2 outcomes: amputation-free survival, defined as the time (in months) to death and/or major amputation, and time to major amputation (above-knee or below-knee) identified by *Current Procedure Terminology* (*CPT*) codes (eTable 1 in Supplement 1). For amputation analysis, death was a competing event.

### Exposures

The exposure of interest was foot and ankle care provided by a podiatrist (physician specialty code 48) within 3 months before study baseline (ie, index DFU diagnosis). It was identified through HCPCS codes (2028F, G0245, G02460, and G0127) and *CPT* codes (11055-57, 11102, 11103, 11305-08, 11420-24, 11426, 11719-21, and 11750) (eTable 1 in Supplement 1).

### Covariates

Covariates included age at kidney failure onset; race; Hispanic ethnicity; sex; underlying cause of kidney disease; functional status, including amputation, inability to ambulate or transfer, need of assistance with daily activities, and being institutionalized; pre-kidney failure nephrology care; and body mass index. Race and ethnicity were considered in the analyses to assess whether there were differences in the use of foot and ankle care by podiatrists before DFU diagnosis. These variables were extracted from the Medical Evidence form at the index diagnosis of kidney failure. Major comorbid conditions evaluated were hypertension, coronary artery disease, congestive heart failure, PAD, cerebrovascular disease, stroke, chronic obstructive pulmonary disease, and other cardiac conditions. Patients were considered to have a comorbidity of interest if they had received at least 1 recorded diagnosis for the condition of interest at either an outpatient or hospital visit. Information on comorbid conditions was ascertained in the 3 months preceding the study baseline. The Charlson Comorbidity Index was used to measure the severity and range of patient comorbid conditions. Dialysis modality (hemodialysis or peritoneal dialysis), duration, and total hospital days during 3 months before the study baseline were also considered. A detailed description of variables, including their definitions and related data sources, is provided in the eMethods and eTable 2 in Supplement 1.

### Statistical Analysis

We calculated bivariate frequencies and percentages to compare baseline characteristics of the podiatry care and nonpodiatry care groups using Pearson χ^2^ tests for categorical variables and *t* tests for continuous variables. Bivariate and adjusted multivariate logistic regression models were used to identify factors associated with receiving foot and ankle care by podiatrists. Odd ratios (ORs) and 95% CIs are reported. The Kaplan-Meier method was used to estimate amputation-free survival probability at 3 years.

To account for the nonrandomized distribution of the 2 groups, inverse probability of treatment weighting (IPTW)–adjusted analyses were used to control for differences in baseline characteristics. Propensity score weighting was used for adjusted analyses, and balance was assessed before and after weighting using absolute standardized mean difference. A value of less than 0.1 was considered balanced (eFigure in Supplement 1). Using IPTW Cox proportional hazards regression, hazard ratios (HRs) for a composite of death and major amputation were estimated for the podiatry care group compared with the no podiatry care group. We observed no departure from the proportional hazards assumption, as indicated by the absence of a significant interaction between the exposure group and time. The presence of competing events is an important consideration in the kidney failure population because of the high mortality risk. Mortality was treated as a competing risk in examining amputations. Specifically, the cumulative incidence curve of major amputation was estimated while accounting for early death as a competing risk. In IPTW multivariate analysis, we adopted a subdistribution hazard model to account for the possible nonindependence of censoring on death as a competing event.

Multiple sensitivity analyses were performed to ensure result robustness. First, a negative control outcome analysis assessed residual confounding, exploring the association between podiatric care and an unrelated outcome—hospitalization rates for pneumonia—as a potential surrogate marker for overall patient care quality. Second, to address lead time bias, we examined time intervals (in days) from study entry (January 1, 2017) to the index DFU diagnosis based on podiatry care receipt. Osteomyelitis or gangrene presence at the index DFU diagnosis, as indicators of ulcer severity, were also compared among study groups. Third, analyses were reiterated using propensity score as an adjustment variable and propensity score matching for 55% of the sample. Fourth, exposure measurement (ankle and foot care by podiatrists) was extended to 12 months before the index DFU diagnosis. All analyses were 2-sided, and *P* < .05 was considered statistically significant. Statistical analyses were completed on June 15, 2023, and an updated analysis was performed on December 11, 2023, using SAS software, version 9.4 (SAS Institute LLC).

## Results

The study cohort included 14 935 adults with kidney failure and newly diagnosed DFUs; 6651 patients (44.5%) were female and 8284 were male (55.4%). Mean (SD) age was 59.3 (12.7) years, and 5291 patients (35.4%) were aged 65 years or older at the initiation of kidney replacement therapy. The mean (SD) duration of dialysis was 5.5 (3.3) years. Among the cohort, 414 (2.7%) were Asian, 5235 (35.0%) were Black/African American, 2652 (17.7%) were Hispanic, and 8745 (58.5%) were White. Other baseline characteristics at dialysis initiation are listed in Table 1.

**Table 1.** Characteristics of Dialysis Patients by Recipient of Foot and Ankle Care by Podiatrists in the 3 Months Before the Index DFU

Characteristic^a^	Patients, No. (%)				
	Total (N = 14 935)		With podiatric care (n = 2736)	Without podiatric care (n = 12 199)	
Demographic characteristics					
Sex					
Male	8284 (55.4)		1547 (56.5)	6737 (55.2)	
Female	6651 (44.5)		1189 (43.5)	5462 (44.8)	
Age, y					
Mean (SD), y	59.3 (12.7)		62.8 (12.1)	58.5 (12.7)	
<50	3253 (21.7)		381 (13.9)	2872 (23.5)	
50 to <65	6391 (42.7)		1084 (39.6)	5307 (43.5)	
≥65	5291 (35.4)		1271 (46.5)	4020 (33.0)	
Race and ethnicity^b^					
Asian	414 (2.7)		77 (2.8)	337 (2.8)	
Black or African American	5235 (35.0)		951 (34.8)	4284 (35.1)	
Hispanic	2652 (17.7)		439 (16)	2213 (18.1)	
White	8745 (58.5)		1664 (60.8)	7081 (58)	
Other	541 (3.6)		44 (1.6)	497 (4.1)	
Dual eligibility of Medicare and Medicaid	6467 (43.4)		1121 (41)	5346 (43.8)	
Clinical characteristics at diagnosis of kidney failure					
BMI					
Mean (SD)	32.1 (8.3)		32.0 (8.1)	32.1 (8.4)	
0 to <25	3011 (20.1)		555 (20.3)	2456 (20.1)	
25 to <30	3866 (25.8)		708 (25.9)	3158 (25.9)	
30 to <35	3389 (22.6)		615 (22.5)	2774 (22.7)	
≥35	4669 (31.2)		858 (31.4)	3811 (31.2)	
Primary cause of kidney failure					
Diabetes	10 897 (72.9)		2011 (73.5)	8886 (72.8)	
Hypertension	2732 (18.2)		500 (18.3)	2232 (18.3)	
Glomerulonephritis	441 (2.9)		65 (2.4)	376 (3.1)	
Other/unknown	865 (5.7)		160 (5.8)	705 (5.8)	
Nephrology care before initiation of dialysis					
Yes	9202 (61.6)		1762 (64.4)	7440 (61)	
No	3277 (21.9)		491 (17.9)	2786 (22.8)	
Unknown	2456 (16.4)		483 (17.7)	1973 (16.2)	
Insulin-dependent diabetes at initiation of dialysis	8916 (59.6)		1660 (60.7)	7256 (59.5)	
Alcohol/drug use	2179 (14.5)		36 (1.3)	200 (1.6)	
Need assistance with activities of daily living	1472 (9.8)		303 (11.1)	1169 (9.6)	
Institutionalized	757 (5.1)		228 (8.3)	529 (4.34)	
Dialysis-related characteristics					
Dialysis modality					
Hemodialysis	13 816 (92.5)		2567 (93.8)	11249 (92.2)	
Peritoneal dialysis	1119 (7.4)		169 (6.2)	950 (7.8)	
Duration of dialysis, y					
Mean (SD)	5.5 (3.3)		5.6 (3.3)	5.5 (3.3)	
<2.5	2676 (17.9)		462 (16.9)	2214 (18.1)	
2.5 to <4	3393 (22.7)		574 (21)	2819 (23.1)	
4 to <6.5	4331 (28.9)		821 (30)	3510 (28.8)	
≥6.5	4535 (30.3)		879 (32.1)	3656 (30)	
Comorbidities and hospitalization^c^^,^^d^					
Hypertension	12 547 (84.0)		2333 (85.3)	10 214 (83.7)	
COPD	2837 (18.9)		553 (20.2)	2284 (18.7)	
CHF	6464 (43.2)		1174 (42.9)	5290 (43.4)	
CVA/TIA	2789 (18.6)		569 (20.8)	2220 (18.2)	
ASHD	1614 (10.8)		284 (10.4)	1330 (10.9)	
Peripheral arterial disease	3429 (22.9)		858 (31.4)	2571 (21.1)	
Coronary artery disease	6840 (45.7)		1320 (48.2)	5520 (45.2)	
Other cardiac conditions	4913 (32.8)		853 (31.2)	4060 (33.3)	
Charlson Comorbidity Index score					
Mean (SD)	6.1 (2.3)		6.4 (2.2)	6.0 (2.3)	
<6	6465 (43.2)		1014 (37.1)	5451 (44.7)	
6 to <8	5163 (34.5)		1028 (37.6)	4135 (33.9)	
8 to <9	1513 (10.1)		335 (12.2)	1178 (9.7)	
≥9	1794 (12.0)		359 (13.1)	1435 (11.8)	
Duration of hospitalization, d					
Mean (SD)	7.9 (13.2)		5.5 (10.3)	8.5 (13.7)	
0	7236 (48.4)		1600 (58.5)	5636 (46.2)	
1 to <10	3622 (24.2)		603 (22)	3019 (24.7)	
≥10	4077 (27.2)		533 (19.5)	3544 (29.1)	

Abbreviations: ASHD, atherosclerotic heart disease; BMI, body mass index (calculated as weight in kilograms divided by height in meters squared); CHF, congestive heart failure; COPD, chronic obstructive pulmonary disease; CVA/TIA, cerebrovascular accident/transient ischemic attack.

^a^ All characteristics were found to be significantly different (*P* < .001) between the groups except for sex, BMI, primary cause of kidney failure, alcohol use, CHF, and COPD. eTable 2 in Supplement 1 provides detailed definition for each characteristic.

^b^ Identification of race and ethnicity was obtained from the Medical Evidence Form; ascertainment of race was made by self-report. The Other group includes American Indian, Alaska Native, Pacific Islander, or Native Hawaiian.

^c^ eTable 1 in Supplement 1 provides specific *International Statistical Classification of Diseases and Related Health Problems, Tenth Revision, Clinical Modification* codes used.

^d^ Both comorbid conditions and hospital days were measured during the 3 months prior to baseline.

### Receipt of Foot and Ankle Care by Podiatrists in the 3 Months Before DFU Diagnosis

The study revealed substantial rates of mortality (44.6%) and major amputation (28.3%) in this high-risk population. Of the cohort, 18.4% (n = 2736) received foot and ankle care by podiatrists within 3 months before the index DFU diagnosis. Patients who received care by podiatrists were more likely to be male, to be older, of White race, receiving pre–kidney failure nephrology care, in need of assistance with daily activities, and institutionalized compared with those who did not. They also had higher Charlson Comorbidity Index scores, had a longer duration of dialysis dependency, a greater likelihood of receiving peritoneal dialysis, and extended hospital stay.

**Figure 2.**
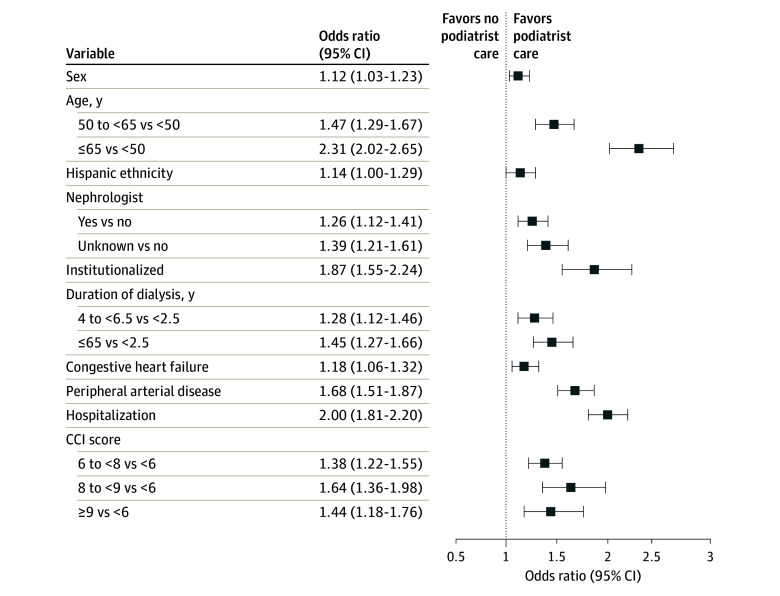
Adjusted Logistic Regression Analysis of Receipt of Foot and Ankle Care by Podiatrists in the 3 Months Before the Diabetic Foot Ulcer Diagnosis CCI indicates Charlson Comorbidity Index.

Adjusted logistic regression analysis (Figure 2) indicated that older age (≥65 vs <50 years: OR, 2.31; 95% CI, 2.02-2.65), institutionalization (OR, 1.87; 95% CI, 1.55-2.24), PAD (OR, 1.68; 95% CI, 1.51-1.87), hospitalization (OR, 2.00; 95% CI, 1.81-2.20), and higher Charlson Comorbidity Index score (8 to <9 vs <6: OR, 1.64; 95% CI, 1.36-1.98) were associated with the likelihood of receiving care by podiatrists. Patients with a longer dialysis duration (≥6.5 vs <2.5 years: OR, 1.45; 95% CI, 1.27-1.66) and receiving pre–kidney failure nephrologist care (OR, 1.26; 95% CI, 1.21-1.41) were also more likely to receive care by podiatrists. Complete logistic regression results can be found in eTable 3 in Supplement 1.

### Associations Between Foot and Ankle Care by Podiatrists With Amputation-Free Survival and Major Amputation

**Figure 3.**
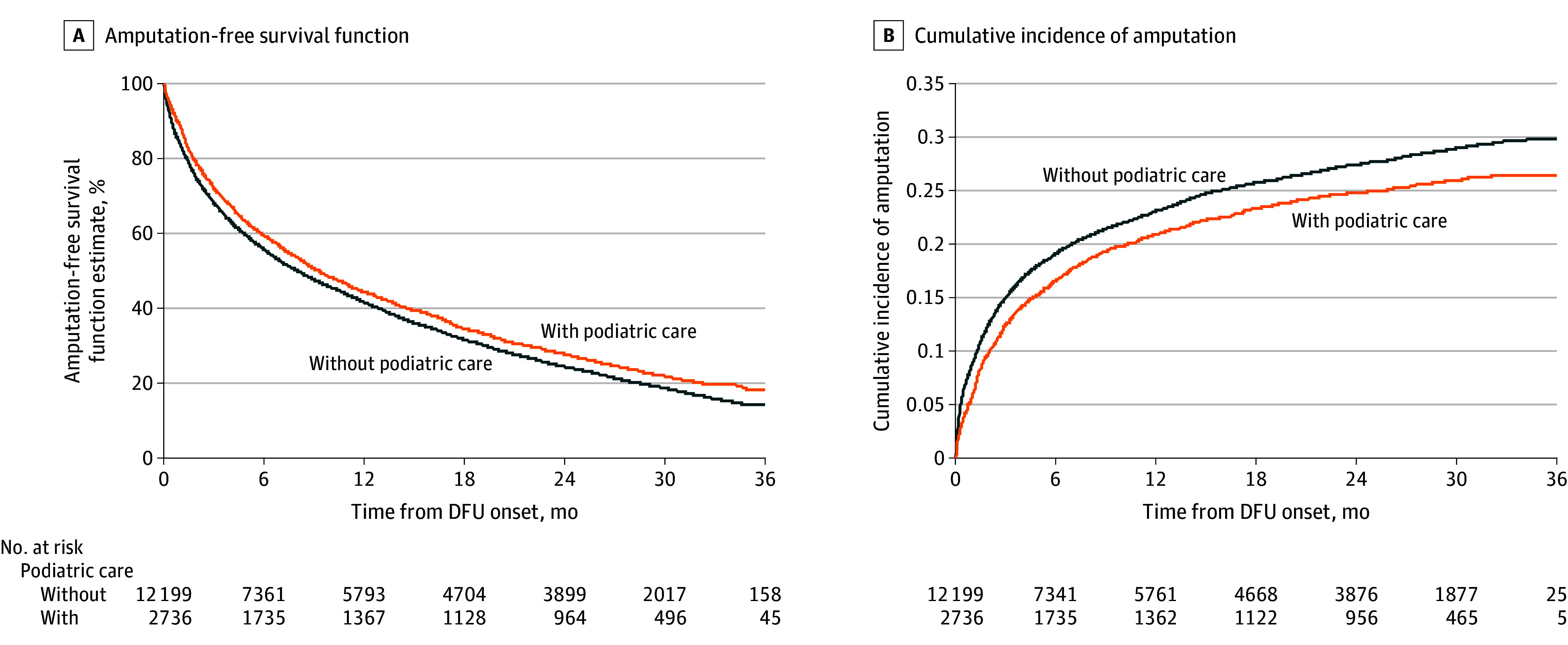
Unadjusted Kaplan-Meier Survival Analysis A, Higher amputation-free survival was observed at 36 months with vs without podiatry care (26.3% vs 22.8%; *P* < .001 by log-rank test). B, Cumulative incidence of amputation alone at 36 months was lower with vs without podiatry care (26.4% vs 29.8%; *P* = .001 by Gray test). DFU indicates diabetic foot ulcer.

At the end of the study follow-up, there were 1217 deaths (44.5%) among patients who received podiatry care (n = 2736) and 5448 deaths (44.7%) among patients who did not (n = 12 199); there were 707 (25.8%) major amputations for those with podiatry care and 3521 (28.9%) for those without podiatry care (Figure 1). Survival probabilities at 36 months were 26.3% vs 22.8% (*P* < .001, unadjusted Kaplan-Meier survival analysis) in the group who received care by podiatrists compared with those who did not (Figure 3, A). Consistently, the cumulative incidence of amputation alone at 36 months was 26.4% in the podiatry care and 29.8% in the no podiatry care groups (*P* = .001 by Gray test) (Figure 3, B).

In unadjusted analysis, receiving care from podiatrists appeared to have a 9% lower likelihood of death or major amputation (HR, 0.91; 95% CI, 0.86-0.95) and a 13% lower likelihood of major amputation alone (HR, 0.87; 95% CI, 0.80-0.93). After adjustment of baseline confounding, the multivariate IPTW model indicated that care by podiatrists was associated with an 11% lower likelihood of the composite of death or major amputation (HR, 0.89; 95% CI, 0.84-0.93; *P* < .001) and a 9% lower likelihood of major amputation alone (HR, 0.91; 95% CI, 0.84-0.99; *P* = .03) (Table 2). eTable 4 in Supplement 1 provides the full results of multivariate analyses.

**Table 2.** Association of Foot and Ankle Care by Podiatrists in the 3 Months Before the Index Diabetic Foot Ulcer Diagnosis With Outcomes

Outcome	No. of total events during follow-up						With vs without podiatric care	
	With podiatric care (n = 2735)			Without podiatric care (n = 12 199)			Unadjusted HR (95% CI)	IP-weighted HR (95% CI)
	Event, No.	% per Patient	% per Patient month	Event, No.	% per Patient	% per Patient month		
Composite of death and amputation	1924	70.3	5.9	8969	73.5	6.6	0.91 (0.86-0.95)	0.89 (0.84-0.93)
Amputation alone^a^	707	25.6	2.1	3521	28.9	2.6	0.87 (0.80-0.94)	0.91 (0.84-0.99)

Abbreviations: HR, hazard ratio; IP, inverse probability.

^a^ HRs were based on subdistribution hazard model.

### Sensitivity Analysis

The sensitivity analysis results addressed concerns regarding residual confounding and lead-time bias. Specifically, pneumonia hospitalization rates were found to be comparable between patients with vs without podiatric care (1.2% vs 1.1%), with no significant difference in the IPTW model (HR, 0.98; 95% CI, 0.92-1.04). Time intervals to DFU diagnosis showed no association with podiatric care, averaging 164 days with care and 162 days without. The podiatric care group had a slightly lower prevalence of osteomyelitis (1.7% vs 1.9%) and gangrene (4.4% vs 4.6%). In the propensity score matching analysis, podiatrist care vs no care was associated with a 13% lower probability of death or amputation (HR, 0.87; 95% CI, 0.82-0.93). Similar results were observed in propensity score–adjusted analysis and when extending the exposure duration from 3 months to 12 months before the index DFU diagnosis.

## Discussion

This claim-based study examined data from a cohort of 14 935 Medicare beneficiaries receiving kidney replacement therapy and newly diagnosed with DFUs to investigate the factors associated with receipt of foot and ankle care by podiatrists and its potential association with outcomes. The study revealed substantial rates of mortality (44.8%) and major amputation (28.3%) in this high-risk population. Only 18.4% of patients received care by podiatrists within the 3 months preceding the DFU diagnosis. Both Kaplan-Meier and adjusted analyses indicated that receipt of foot and ankle care by podiatrists was associated with improvement in 3-year amputation-free survival, a reduced risk of major amputation, and a lower risk of the composite outcome of death and/or major amputation. These findings suggest that preemptive foot and ankle care by podiatrists has the potential to offer benefits in reducing limb loss in high-risk patients with kidney failure and diabetes who are receiving dialysis.

Patients receiving dialysis have a disproportionate burden of peripheral neuropathy, PAD, and diabetes-related foot complications compared with individuals at earlier stages of chronic kidney disease.^13^ Diabetic peripheral neuropathy is prevalent in up to 90% of patients with advanced kidney disease,^24^ and the prevalence of PAD ranges from 23% to 46% in patients receiving dialysis, in contrast to 7% to 24% in those with chronic kidney disease.^25^ In addition, dialysis itself is an independent risk factor for limb loss in patients with both PAD and DFUs.^12^ Hospitalizations related to diabetic foot complications accounted for a higher proportion (33%) of amputation in patients with kidney failure and DFUs compared with nondialysis diabetic populations (14%).^4^ Furthermore, this cohort of 51 362 patients with kidney failure and DFUs exhibited a significantly higher mortality rate (71% over 3 years of follow-up)^4^ compared with usual cancer mortality and patients with kidney failure without diabetic foot complications.^26^

Given the substantial morbidity and mortality associated with DFUs, coupled with poor outcomes from revascularization in patients with kidney failure, care guidelines recommend regular foot screening and preventive care for individuals receiving dialysis.^16,25^ The 2023 International Working Group on Diabetic Foot guidelines categorize patients with kidney failure and peripheral neuropathy or PAD history as category 3 and recommend quarterly foot screenings.^16^ Implementing diabetes education and care management, including regular foot examinations, has been shown to improve glycemic control and reduce amputations in patients with kidney failure.^27^ In a study by Pernat et al,^28^ monthly foot examinations conducted by dialysis nurses at hemodialysis facilities resulted in a 17% reduction in major amputation rates.

Our study findings align with prior evidence underscoring the importance of care by podiatrists for patients with diabetes before and after the onset of DFUs. For example, Medicare beneficiaries with DFUs who received care from podiatrists and dedicated lower extremity complication specialists were less likely to undergo amputation compared with those treated by nondedicated specialists.^29^ A meta-analysis further highlighted that multidisciplinary care, including podiatrists, was associated with a 31% decreased risk of any amputation and a 55% lower risk of major amputation.^17^ Additionally, a study by Gibson et al^18^ reported that care provided by podiatrists in the year preceding a DFU diagnosis was associated with decreased risks of limb loss and hospitalizations. Taken together, these findings, including those from our study, underscore the potential of care by podiatrists to mitigate the heightened amputation risks faced by patients with diabetes who are receiving dialysis.

In our study, patients undergoing dialysis who received foot and ankle care by podiatrists exhibited distinct characteristics. Specifically, they tended to be older, presented with a higher burden of comorbidities, and needed assistance with activities of daily living, compared with those who did not receive such care. A substantial proportion of at-risk patients with kidney failure, accounting for more than 80% of the study cohort, did not receive such care within the 3 months leading up to their DFU diagnosis. This observation highlights a critical gap in the delivery of preventive measures for a population inherently susceptible to diabetes-related foot complications. Previous studies have highlighted wide variations in routine preventive podiatry care coverage across insurance types, states, and even within states depending on Medicaid Advantage–Managed Care programs.^30,31^ Addressing these challenges and the policy implications of expanded coverage are beyond this article’s scope, requiring further comprehensive studies. An understanding of the underlying patient-level and health care system–level factors that contribute to this gap can guide the development of targeted interventions to ensure more individuals at risk for DFUs receive appropriate preemptive foot and ankle care.

### Limitations

This study has several important limitations. First, it relied on administrative claims data and the use of *ICD-10-CM* codes for identifying the study cohort, introducing inherent limitations associated with data accuracy and completeness. Second, due to data constraints, information regarding the frequency and content of podiatry visits, as well as foot and ankle care from nonpodiatry clinicians, was not captured. The absence of these details hampers the ability to examine the optimal nature and extent of foot and ankle care. Third, the study cohort focused on individuals who developed DFUs without a recent history of amputation or DFUs, which could potentially limit the generalizability of these findings to broader chronic kidney disease populations. Fourth, the relatively short duration of follow-up may limit our capability to draw definitive conclusions regarding the long-term efficacy of care provided by podiatrists within this population. We also did not examine how foot and ankle care by podiatrists could enhance the likelihood of kidney transplant in patients with kidney failure by reducing the presence of open wounds.

## Conclusions

In this comprehensive analysis of a national cohort comprising Medicare beneficiaries with kidney failure and DFUs, the receipt of foot and ankle care provided by podiatrists within the 3 months preceding a DFU diagnosis was associated with reduction in the risks of major amputation and death among patients with kidney failure who were receiving dialysis. While future fully powered studies are needed to further support these findings, these results suggest positive potential benefits for preventive foot and ankle care to mitigate complications in this high-risk population.

## Notes

Supplement 1. **eTable 1.** Diagnosis and Procedure Codes **eMethods.** Data Sources, Variable Constructions, and Missing Data **eTable 2.** Independent Study Variables and Related Data Sources **eTable 3.** Association Between Study Covariates and the Likelihood of Receiving Foot and Ankle Care by Podiatrists in the 3-Months Prior to Index DFU **eTable 4.** Association for Receipt of Foot and Ankle Care by Podiatrists and Outcomes Using Inverse Probability of Treatment Weighting (IPTW) Cox Regression Analysis for Composite Outcomes of Death and/or Amputation and IPTW Competing Risk Analysis for Cumulative Incidence of Major Amputation **eFigure.** Propensity Score Weighting for Adjusted Analyses

Supplement 2.
Data Sharing Statement
