# Supplementary material for: Foot and Ankle Care by Podiatrists and Amputations in Patients With Diabetes and Kidney Failure
Source: JAMA Netw Open. 2024 Mar 1;7(3):e240801. doi: 10.1001/jamanetworkopen.2024.0801 (PMC10907919; doi:10.1001/jamanetworkopen.2024.0801)
Supplement: Supplement 1. — eTable 1. Diagnosis and Procedure Codes eMethods. Data Sources, Variable Constructions, and Missing Data eTable 2. Independent Study Variables and Related Data Sources eTable 3. Association Between Study Covariates and the Likelihood of Receiving Foot and Ankle Care by Podiatrists in the 3-Months Prior to Index DFU eTable 4. Association for Receipt of Foot and Ankle Care by Podiatrists and Outcomes Using Inverse Probability of Treatment Weighting (IPTW) Cox Regression Analysis for Composite Outcomes of Death and/or Amputation and IPTW Competing Risk Analysis for Cumulative Incidence of Major Amputation eFigure. Propensity Score Weighting for Adjusted Analyses [file jamanetwopen-e240801-s001.pdf]

Supplementary Online Content

Tan TW, Caldwell B, Zhang Y, Kshirsagar O, Cotter DJ, Brewer TW. Foot and ankle care by podiatrists and amputations in patients with diabetes and kidney failure. *JAMA Netw Open*. 2024;7(3):e240801. doi:10.1001/jamanetworkopen.2024.0801

eTable 1. Diagnosis and Procedure Codes

eMethods. Data Sources, Variable Constructions, and Missing Data

eTable 2. Independent Study Variables and Related Data Sources

eTable 3. Association Between Study Covariates and the Likelihood of Receiving Foot and Ankle Care by Podiatrists in the 3-Months Prior to Index DFU

eTable 4. Association for Receipt of Foot and Ankle Care by Podiatrists and Outcomes Using Inverse Probability of Treatment Weighting (IPTW) Cox Regression Analysis for Composite Outcomes of death and/or Amputation and IPTW Competing Risk Analysis for Cumulative Incidence of Major Amputation

eFigure. Propensity Score Weighting for Adjusted Analyses

This supplementary material has been provided by the authors to give readers additional information about their work.

© 2024 Tan TW et al. *JAMA Network Open.*

eTable 1. Diagnosis and Procedure Codes

Variables	Code
HCPCS codes for foot and ankle care	11721,11720,11056, G0127,11719,11055,11730,11057,4040F, A5500
Major amputation	27590, 27591, 27592, 27594, 27596, 27598, 27880, 27881, 27882, 27884, 27886, 27888, 27889
Minor amputation	28825, 28820, 28810, 28805, 28122, 28800
Ulcer diagnosis	L97901, L97902, L97903, L97904, L97905, L97906, L97908, L97909, L97911, L97912, L97913, L97914, L97915, L97916, L97918, L97919, L97921, L97922, L97923, L97924, L97925, L97926, L97928, L97929, L97101, L97102, L97103, L97104, L97105, L97106, L97108, L97109, L97111, L97112, L97113, L97114, L97115, L97116, L97118, L97119, L97121, L97122, L97123, L97124, L97125, L97126, L97128, L97129, L97201, L97202, L97203, L97204, L97205, L97206, L97208, L97209, L97211, L97212, L97213, L97214, L97215, L97216, L97218, L97219, L97221, L97222, L97223, L97224, L97225, L97226, L97228, L97229, L97301, L97302, L97303, L97304, L97305, L97306, L97308, L97309, L97311, L97312, L97313, L97314, L97315, L97316, L97318, L97319, L97321, L97322, L97323, L97324, L97325, L97326, L97328, L97329, L97401, L97402, L97403, L97404, L97405, L97406, L97408, L97409, L97411, L97412, L97413, L97414, L97415, L97416, L97418, L97419, L97421, L97422, L97423, L97424, L97425, L97426, L97428, L97429, L97501, L97502, L97503, L97504, L97505, L97506, L97508, L97509, L97511, L97512, L97513, L97514, L97515, L97516, L97518, L97519, L97521, L97522, L97523, L97524, L97525, L97526, L97528, L97529, L97801, L97802, L97803, L97804, L97805, L97806, L97808, L97809, L97811, L97812, L97813, L97814, L97815, L97816, L97818, L97819, L97821, L97822, L97823, L97824, L97825, L97826, L97828, L97829, L98499
Diabetes	E11.xxx

**Comorbidity at baseline**	**ICD-10-CM Code**
Hypertension	I10, I110, I119, I120, I129, I130, I1310, I1311, I132, I150, I151, I152, I158, I159
Chronic obstructive pulmonary disease (COPD)	J410, J411, J418, J42, J430, J431, J432, J438, J439, J440, J441, J449, J982, J983
Heart failure (CHF)	A18.84, I09.81, I11.0, I13.0, I13.2, I40.0-I43, I50.1- I50.9, Z48.21, Z48.280, Z94.1, Z94.3, I50.20-I50.23, I50.40-I50.43
Cerebrovascular accident/ transient ischemic attack (CVA/TIA)	G45.0-G45.2, G45.4-G46.8, I60.00-I66.9, I67.1, I67.2, I67.4-I67.82, I67.841-I69.998
Atherosclerotic heart disease (ASHD)	I20, I21, I22, I23, I240, I241, I248, I249, I25, I510, I512
Other cardiac disease	I050, I051, I052, I058, I059, I060, I061, I062, I068, I069, I070, I071, I072, I078, I079, I080, I081, I082, I083, I088, I089, I091, I300, I301, I308, I309, I310, I311, I312, I313, I314, I318, I319, I32, I330, I339, I340, I341, I342, I348, I349, I350, I351, I352, I358, I359, I360, I361, I362, I368, I369, I370, I371, I372, I378, I379, I38, I39, I400, I401, I408, I409, I41, I511, I513, I514, I515, I517, I518, I519, I52, I970, I971

© 2024 Tan TW et al. *JAMA Network Open.*

Diabetes at initiation of dialysis	Medical Evidence File
Peripheral artery disease (PAD)	E08.51, E08.52, E09.51, E09.52, E10.51, E10.52, E11.51, E11.52, E13.51, E13.52, I67.0, I70.0-I74.9, I77.0-I77.9, I79.0-I79.8, K55.0, K55.1, K55.8, K55.9, M31.8, M31.9
Coronary artery disease (CAD)	I12.00-I22.9, I24.0-I25.9, Z95.1, Z95.5, Z98.61

© 2024 Tan TW et al. *JAMA Network Open.*

eMethods. Data sources, variable constructions, and missing data

1) **Data sources.**

Standard analytical files (SAF's) collected and maintained by the United States Renal Data System (USRDS) were used to obtain patient clinical history and characteristics. This database incorporates extensive baseline and follow-up demographic and clinical data on ESRD patients whose care is covered by the Medicare program. Individual patient demographics were obtained from the PATIENTS file in the USRDS Core CD. Patient clinical factors at the initiation of ESKD were extracted from Medical Evidence file (CMS 2728 form). Patient co-morbid conditions and severity of illness were obtained from the institutional claims' files. Diagnosis and procedures were identified from both outpatient, inpatient, and physician claims. Physician claims were also used to extract physician Specialty. The variables included in the USRDS SAF's, as well as the data source, collection methods, and validation studies, are listed at the USRDS website, https://www.niddk.nih.gov/about-niddk/strategic-plans-reports/usrds. We used the USRDS standard analytic files as of calendar years between 2016 and 2019 for the analysis reported in the manuscript.

2) Variable construction

For study outcomes, death information was obtained from Patients file. Major amputation (above-knee, below-knee) was identified by CPT codes (eTable 1) from inpatient, outpatient, and physician/supplier claim files. eTable 2 below lists the descriptions of independent variables included in the analyses.

3) Missing data

Variables with missing values include drug/alcohol use, diabetes status, institutionalization status, primary cause of renal failure, and pre-dialysis nephrology care. Specifically, for variables such as drug and alcohol use, diabetes (insulin use) status, and institutionalization status, the extent of missing data is minimal, comprising only 0.6%. This missing data is evenly distributed across the groups. As for the causes of renal failure, there were a total of 181 cases with unknown

© 2024 Tan TW et al. *JAMA Network Open.*

causes, with 141 (1.2%) in the group without podiatry care and 40 (1.5%) in the group with podiatry care. Predominantly, missing values were related to pre-dialysis nephrology care, with 764 cases missing and 1,692 unknowns, totaling 2,456 (16.2% in the without-podiatry-care group and 17.7% in the with-podiatry-care group).

In our main analysis, we treated missing cases of pre-dialysis nephrology care as a distinct category in our models, facilitating a complete case analysis. In sensitivity analysis, we excluded missing values and repeating the analysis. Results reported in the manuscript remained essentially unchanged with the exclusion of the missing data category.

© 2024 Tan TW et al. *JAMA Network Open.*

eTable 2. Independent study variables and related data sources

Variables	*Description*	*Data Source*
Demographics		
Age at first ESRD service	Categorized into 3 groups: < 50, 50 - < 65, and ≥65	USRDS Patients file
Gender	Male/Female	USRDS Patients file
Race	Asian, Black/African American<br>Other (American Indian, Alaska native, Pacific Islander, or native Hawaiian) and White	USRDS Patients file
Hispanic ethnicity	yes/no	USRDS Patients file
Dual eligibility of Medicare and Medicaid	yes/no	USRDS Medical Evidence file
Clinical characteristics at initiation of ESRD		
Primary cause of ESKD*	Diabetes/Hypertension/ Glomerulonephritis/Other	USRDS Patients file
Body mass index (kg/m^2)	Categorized into 4 groups: 0 - < 25, 25 - < 30, 30 - < 35, ≥ 35	USRDS Medical Evidence data
Nephrology care before initiation of dialysis*	yes/no	USRDS Medical Evidence file
Diabetes on insulin at initiation of dialysis*	yes/no	USRDS Medical Evidence file
Alcohol/Drug Use*	yes/no	USRDS Medical Evidence file
Need assistance with activities of daily living*	yes/no	USRDS Medical Evidence file
Institutionalized*	yes/no	USRDS Medical Evidence file
Dialysis related characteristics		
Dialysis modality	Hemodialysis/Peritoneal dialysis	USRDS outpatient dialysis claims file
Duration of dialysis, y	Time between the initiation of dialysis and study baseline; categorized into 4 groups: < 2.5, 2.5 - < 4, 4 - < 6.5, and ≥ 6.5	USRDS Patients file and claims file
Comorbidities and hospitalization		
Hypertension	yes/no	USRDS institutional claims and physician/supplier claims
Chronic obstructive pulmonary disease (COPD)	yes/no	USRDS institutional claims and physician/supplier claims
Congestive heart failure (CHF)	yes/no	USRDS institutional claims and physician/supplier claims

© 2024 Tan TW et al. *JAMA Network Open.*

Variables	*Description*	*Data Source*
Cerebrovascular accident/ transient ischemic attack (CVA/ TIA)	yes/no	USRDS institutional claims and physician/supplier claims
Atherosclerotic heart disease (ASHD)	yes/no	USRDS institutional claims and physician/supplier claims
Peripheral arterial disease (PAD)	yes/no	USRDS institutional claims and physician/supplier claims
Coronary artery disease (CAD)	yes/no	USRDS institutional claims and physician/supplier claims
Other cardiac conditions	yes/no	USRDS institutional claims and physician/supplier claims
Charlson Comorbidities Index (CCI) Score	< 6, 6 - < 8, 8 - < 9, and ≥ 9	USRDS institutional claims and physician/supplier claims
Duration of hospitalization, days	0, 1 - < 10, and ≥ 10	USRDS institutional claims and physician/supplier claims

© 2024 Tan TW et al. *JAMA Network Open.*

eTable 3. Association between study covariates and the likelihood of receiving foot and ankle care by podiatrists in the 3-months prior to index DFU.

Variables	*OR*	*LL*	*UL*
**Patient Demographics**			
Age (ref < 50)			
50 - < 65	1.47	1.29	1.67
≥ 65	2.31	2.02	2.65
Gender (ref Female)	1.12	1.03	1.23
Race (ref White)			
Black/African American	0.96	0.87	1.07
Asian	0.86	0.66	1.12
Other/unknown	0.41	0.30	0.57
Hispanic ethnicity (ref Yes)	1.14	1.00	1.29
Dual eligibility of Medicare and Medicaid (ref No)	1.01	0.92	1.10
**Clinical characteristics at initiation of ESRD (2728 form)***			
BMI (ref < 25)			
25 - < 30	0.97	0.85	1.10
30 - < 35	0.97	0.85	1.11
≥ 35	1.04	0.92	1.18
Primary Cause of renal failure detailed group (ref Diabetes)			
Hypertension	0.97	0.86	1.09
Glomerulonephritis	0.90	0.69	1.19
Other/Unknown	0.93	0.77	1.12
Nephrologist (ref No)			
Yes	1.26	1.12	1.41
Unknown	1.39	1.21	1.61
Diabetes on insulin at initiation of dialysis (ref No)	1.05	0.96	1.16
Alcohol/Drug Use (ref No)	0.97	0.67	1.41
Need assistance with activities of daily living (ref No)	0.94	0.80	1.09
Institutionalized (ref No)	1.87	1.55	2.24
**Dialysis related characteristics**			
Modality at baseline (ref hemodialysis)			
Peritoneal dialysis	0.88	0.73	1.05
Duration of dialysis ref (< 2.5 yrs)			
2.5 yrs - < 4 yrs	1.02	0.89	1.18
4 yrs - < 6.5 yrs	1.28	1.12	1.46
≥ 6.5 yrs	1.45	1.27	1.66
**Comorbidities in Baseline**			
Hypertension (ref No)	1.11	0.97	1.26
Chronic obstructive pulmonary disease (COPD) (ref No)	1.01	0.90	1.14
Congestive heart failure (ref Yes)	1.18	1.06	1.32

© 2024 Tan TW et al. *JAMA Network Open.*

Cerebrovascular accident/ transient ischemic attack (CVA/ TIA) (ref No)	1.06	0.94	1.19
Atherosclerotic heart disease (ASHD) (ref No)	0.97	0.83	1.13
Peripheral arterial disease (ref No)	1.68	1.51	1.87
Coronary artery disease (CAD) (ref No)	1.02	0.93	1.13
Other cardiac conditions (ref No)	0.92	0.83	1.02
Charlson index score (ref < 6)			
6 - < 8	1.38	1.22	1.55
8 - < 9	1.64	1.36	1.98
≥ 9	1.44	1.18	1.76
Hospitalization (ref No)	2.00	1.81	2.20

OR: odds ratio; LL: lower limit of 95% confidence interval; UL: upper limit of 95% confidence interval

© 2024 Tan TW et al. *JAMA Network Open.*

eTable 4. Association for receipt of foot and ankle care by podiatrists and outcomes using inverse probability of treatment weighting (IPTW) Cox Regression analysis for composite outcomes of death and/or amputation and IPTW competing risk analysis for cumulative incidence of major amputation

**eTable S4a:** Hazard ratio for a composite of death and/or major amputation based on IPTW Cox Regression				
**Variable**	**Hazard Ratio**	**95% Lower Limit**	**95% Upper Limit**	**Probability > Chi-square**
Foot and ankle care by podiatrists (ref No)	0.89	0.84	0.93	<.0001
**Patient Demographics**				
Gender (ref Male)	0.86	0.82	0.89	<.0001
Age*	1.02	1.01	1.02	<.0001
Race of patient (ref White)				
Black/African American	0.89	0.85	0.93	<.0001
Asian	0.82	0.72	0.92	0.0008
Other/ unknown	0.85	0.76	0.94	0.0021
Hispanic ethnicity (ref No)	0.82	0.77	0.87	<.0001
Dual eligibility of Medicare and Medicaid (ref No)				
Yes	1.04	0.99	1.11	0.1432
**Clinical characteristics at initiation of end-stage kidney disease (2728 form) ***				
Body mass index (BMI)				
BMI*	1.00	0.98	1.01	0.5293
Primary Cause of renal failure detailed group (ref Diabetes)				
Hypertension	0.98	0.93	1.03	0.3542
Glomerulonephritis	0.96	0.86	1.08	0.5217
Other/Unknown	0.96	0.88	1.04	0.2681
Nephrologist at initiation of dialysis (ref Yes)				
No	1.01	0.96	1.06	0.7425
Unknown	1.02	0.96	1.09	0.4585
Diabetes on insulin at initiation of dialysis (ref No)	1.10	1.05	1.14	<.0001
Alcohol/ Drug Use (ref No)	1.06	0.92	1.23	0.4133
Need assistance with activities of daily living (ref No)	0.97	0.91	1.04	0.3948
Institutionalized (ref No)				
Yes	1.03	0.95	1.12	0.4915
**Dialysis related characteristics**				
Modality at baseline (ref Hemodialysis)				
Peritoneal dialysis	1.28	1.19	1.39	<.0001
Duration of dialysis				
Dialysis duration*	1.05	1.00	1.09	0.0417
**Comorbidities in Baseline**				

© 2024 Tan TW et al. *JAMA Network Open.*

Hypertension (ref No)	0.87	0.82	0.92	<.0001
Chronic obstructive pulmonary disease (COPD) (ref No)	1.07	1.02	1.13	0.0109
Congestive heart failure (CHF) (ref No)	1.10	1.05	1.16	0.0001
Cerebrovascular accident/Transient ischemic attack (CVA/TIA) (ref No)	1.05	0.99	1.11	0.0783
Atherosclerotic heart disease (ASHD) (ref No)	1.00	0.94	1.07	0.9075
Peripheral arterial disease (PAD) (ref No)	1.12	1.06	1.17	<.0001
Coronary artery disease (CAD) (ref No)	1.1	1.06	1.15	<.0001
Other cardiac conditions (ref No)	1.00	0.95	1.04	0.8173
Charlson Index Score				
CCI*	0.99	0.96	1.03	0.662
Number of days patient is hospitalized				
Total hospital days*	1.06	1.05	1.06	<.0001

eTable S4b: Hazard ratio for major amputation based on IPTW competing risk analysis

**Variable**	**Hazard Ratio**	**95% Lower Limit**	**95% Upper Limit**	**Probability > Chi-square**
Foot and ankle care by podiatrists (ref No)	0.91	0.84	0.99	0.0251
**Patient Demographics**				
Gender (ref Male)	0.69	0.65	0.74	<.0001
Age*	1.01	1.01	1.02	0.0007
Race of patient (ref White)				
Black/African American	1.35	1.25	1.45	<.0001
Asian	0.83	0.67	1.03	0.0967
Other/unknown	0.98	0.83	1.16	0.8065
Hispanic ethnicity (ref No)	1.08	0.99	1.19	0.077
Dual eligibility of Medicare and Medicaid (ref No)				
Yes	0.99	0.93	1.06	0.7489
**Clinical characteristics at initiation of ESKD (2728 form)***				
Body Mass Index (BMI)				
BMI*	1.02	1.00	1.05	0.1062
Primary cause of renal failure detailed group (ref Diabetes)				
Hypertension	0.89	0.82	0.97	0.011
Glomerulonephritis	0.88	0.72	1.07	0.1906
Other/Unknown	0.74	0.63	0.87	0.0002
Nephrologist at initiation of dialysis (ref Yes)				
No	0.93	0.86	1.00	0.0506

© 2024 Tan TW et al. *JAMA Network Open.*

Unknown	0.97	0.88	1.07	0.5321
Diabetes on insulin at initiation of dialysis (ref No)	1.12	1.04	1.2	0.0021
Alcohol/Drug Use (ref No)	0.92	0.72	1.18	0.5057
Need assistance with activities of daily living (ref No)	0.91	0.81	1.02	0.1052
Institutionalized (ref No)				
Yes	0.88	0.74	1.04	0.1259
**Dialysis related characteristics**				
Modality at baseline (ref hemodialysis)				
Peritoneal dialysis	1.13	1.01	1.27	0.0334
Duration of dialysis				
Dialysis duration*	0.92	0.86	0.99	0.0206
**Comorbidities in baseline**				
Hypertension (ref No)	0.82	0.75	0.9	<.0001
Chronic obstructive pulmonary disease (COPD) (ref No)	0.81	0.73	0.89	<.0001
Congestive heart failure (CHF) (ref No)	0.88	0.82	0.96	0.0026
Cerebrovascular accident/Transient ischemic attack (CVA/TIA) (ref No)	0.99	0.90	1.08	0.7584
Atherosclerotic heart disease (ASHD) (ref No)	0.99	0.88	1.1	0.8261
Peripheral arterial disease (PAD) (ref No)	1.35	1.25	1.47	<.0001
Coronary artery disease (CAD) (ref No)	1.09	1.02	1.18	0.0136
Other cardiac conditions (ref No)	0.8	0.74	0.87	<.0001
Charlson Comorbidities Index (CCI) Score				
CCI*	1.02	0.97	1.08	0.4437
No of days patient is hospitalized				
Total hospital days*	1.06	1.05	1.07	<.0001

*Different splines for these continuous covariates were created and used to capture potential non-linear relationships with the outcome variable, allowing for flexibility in modeling the non-linear effects of each variable independently. These spline variables were created using Frank Harrell's SAS Macro %RCSPLINE. Specific spline placements are listed in the table below (also included in supplemental files). The choice of splines was based on percentile distribution of the observed data to capture potential non-linearities. We considered various spline options and experimented with different knot placements to optimize model fit while avoiding overfitting.

Age		Total inpatient days		CCI		BMI		Dialysis duration	
Percentiles	Values	Percentiles	Values	Percentiles	Values	Percentiles	Values	Percentiles	Values
5	37	5	3	5	3	5	21	5	2
10	43	25	6	25	5	25	26	25	3
25	54	50	10	75	8	50	31	75	6
75	66	75	20	95	11	75	39	95	11
95	80	95	55			95	46		

© 2024 Tan TW et al. *JAMA Network Open.*

eFigure. Propensity score weighting for adjusted analyses.

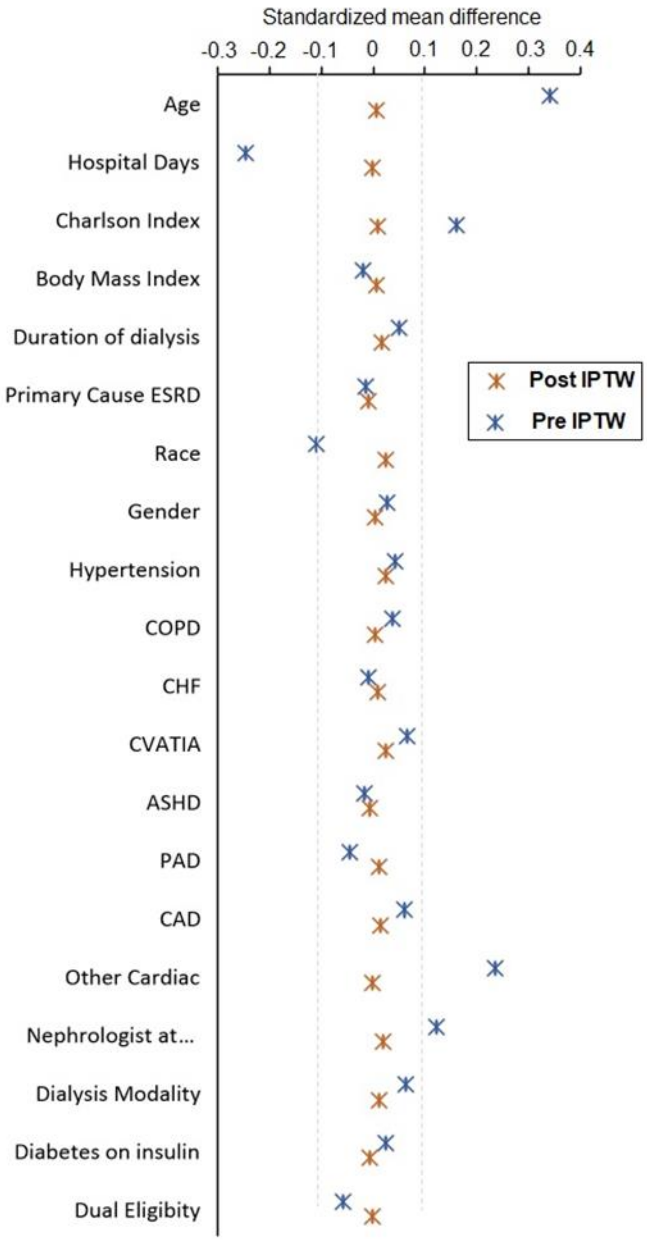

Balance of propensity score weighting was assessed before and after weighting using absolute standardized mean difference. A value of <0.1 was considered balance.

© 2024 Tan TW et al. *JAMA Network Open.*
