# Supplementary material for: Foot and Ankle Care by Podiatrists and Amputations in Patients With Diabetes and Kidney Failure
Source: JAMA Netw Open. 2024 Mar 1;7(3):e240801. doi: 10.1001/jamanetworkopen.2024.0801 (PMC10907919; doi:10.1001/jamanetworkopen.2024.0801)
Supplement: Supplement 2. — Data Sharing Statement [file jamanetwopen-e240801-s002.pdf]

Data Sharing Statement

Tan. Foot and Ankle Care by Podiatrists and Amputations in Patients With Diabetes and Kidney Failure. *JAMA Netw Open*. Published March 01, 2024.
doi:10.1001/jamanetworkopen.2024.0801

Data
Data available: Yes
Data types: Deidentified participant data
How to access data: Our data source was the United State Renal Data System (USRDS). USRDS maintains an independent database encompassing diagnoses, demographic characteristics, biochemical data, dialysis claims, and information on treatment and payer histories, hospitalization events, deaths, healthcare services, and providers for ESRD patients. The data used for this study can be shared upon request and approval from the USRDS.
Email: tze-woei.tan@med.usc.edu
When available: With publication

Supporting Documents
Document types: None

Additional Information
Who can access the data: Researchers whose proposed use of the data has been approved by USRDS.
Types of analyses: Researchers whose proposed use of the data has been approved by USRDS.
Mechanisms of data availability: After approval of proposal.
